# Maternal attachment representation, the risk of increased depressive symptoms and the influence on children’s mental health during the SARS-CoV-2-pandemic

**DOI:** 10.1007/s10826-021-02162-4

**Published:** 2021-11-17

**Authors:** Franziska Köhler-Dauner, Anna Buchheim, Katherina Hildebrand, Inka Mayer, Vera Clemens, Ute Ziegenhain, Jörg M. Fegert

**Affiliations:** 1 Department of Child and Adolescent Psychiatry/Psychotherapy, University Hospital of Ulm Medical University of Ulm, Steinhövelstraße 5, 89075 Ulm, Germany; 2 Institut für Psychologie, Universität Innsbruck, Innrain 52 f, 6020 Innsbruck, Austria

**Keywords:** SARS-CoV-2-pandemic, Parental childhood maltreatment (CM), Maternal attachment representation, Depression symptoms

## Abstract

The social distancing measures and the related closure of education institutions have confronted young families, in particular, with various challenges. Additional risk factors such as an insecure or even unresolved maternal attachment representation may affect mental health of mothers and their children in times of increased stress such as during the ongoing pandemic. We aimed to analyze the interplay between maternal attachment representation and mother’s and children’s mental health before and during the SARS-CoV-2-pandemic. 91 mothers completed a “SARS-CoV-2 pandemic survey” examining the pandemic-related stress of their families including their own depressive symptomology and their children’s mental health. Our mediation analysis demonstrates that the mothers’ depressive symptomology significantly and fully mediated the relationship between maternal attachment representations and children’s mental health during the pandemic. In contrast, the indirect effect of the maternal attachment representation on children’s mental health before the pandemic through the depressive symptoms experienced by the mothers before the pandemic did not reach significance alongside the total and direct effect. The quality of the maternal attachment representation, promoted by childhood maltreatment, seems to be one relevant risk factor for the mothers’ and children’s mental health during a stressful time like a pandemic. The risk for mothers to develop depressive symptoms in times of a pandemic is significantly influenced by their current representation of previous attachment experiences. In addition, the mental well-being of mothers showed a considerable influence on the children’s mental health during a pandemic. The results underline the necessity to consider unique needs of family members and to offer specific support in the current crisis focusing on attachment issues.

## Highlights

- Parental childhood maltreatment: Experiences of CM are more likely to develop an insecure and unresolved attachment representation in adulthood and this is a risk factor for increased depressive symptoms.
- Maternal attachment representation: Additional risk factors such as an insecure or even unresolved maternal attachment representation may affect mental health of mothers and their children in times of increased stress such as during the ongoing pandemic.
- SARS-CoV-2-pandemic: The social distancing measures and the related closure of education institutions during the pandemic have confronted young families, in particular, with various challenges.

## Introduction

The current severe acute respiratory syndrome coronavirus type 2 (SARS-CoV-2) pandemic has been imposing numerous restrictions and challenges for everyone in the world for over one year. Especially young families have been faced with various challenges and measures. They were faced with recommendations for increasing physical distance, sudden closure of schools and childcare, the loss of community programs and jobs, increasing pressure from recession or unemployment, home schooling, lack of social support among others, from grandparents, which require them all to find solutions to the emerging problems (Fegert et al., 2020; Halvorsen et al., 2020; Johns Hopkins University, 2021). “Social distancing” is also confronting the little ones in our society with considerable challenges. With the additional closure of organized leisure activities in clubs, churches and other institutions as well as the impossibility of informal face-to-face meetings for children and young people, a new life situation arises for them where crucial and development-relevant parameters of everyday life suddenly break away. We might assume that the ongoing pandemic will have social, emotional and cognitive effects that we do not yet know in detail but can foresee (Feinberg et al., 2021).

A relevant protective factor in times of a pandemic seems to be a stable parental home. Numerous studies have already shown that especially in times of stress and uncertainty triggered by a pandemic, especially young children urgently need a secure and stable family environment (Schofield et al., 2013; Flouri et al., 2015; Hostinar et al., 2014; Yue et al., 2020). In addition, previous economic recessions have shown that factors like unemployment, decreasing income, excessive debt and parental history of psychological stress pose serious threats to a family’s mental health. This has been shown in terms of a decrease in mental well-being as well as increasing rates of various mental disorders, parental substance-related disorders or suicidal behavior (Feinberg et al., 2021; Fegert et al., 2020; Wu et al., 2020; Frasquilho et al., 2016; Haw et al., 2015). Spinelli and colleagues reported in a current study that the health risks and fears associated with SARS-CoV-2 have an impact on the parental mental health and their perception of stress and that these changes negatively affect the children’s well-being (Spinelli et al., 2020; Pierce et al., 2020). Several studies suggest a strong link between parental mental health and children’s mental well-being under pre-pandemic normal conditions (Weissman et al., 2006; Beardslee et al., 2011; Reger et al., 2020; Roos et al., 2021), expressed through the development of internalizing problems (Fanti et al., 2008), externalizing problems (Gross et al., 2008) as well as emotional adjustment (van der Valk et al., 2007). We may consider that the importance of parental mental health is evident and in fact, not all parents are able to be particularly stress-resistant and organized in times of a pandemic in order to provide a safe and protective environment for their children.

One factor that may be of importance on how parents deal with pandemic-associated stressors seems to be the parents’ own mental health. Agid et al. (2000) suggest the experience of stressful early life experiences as a relevant predictor increasing parental mental health problems. Stressful early life experiences like experiences of childhood maltreatment (CM), abuse or neglect relate to further characteristics such as adult attachment representation (Morton & Browne, 1998; Finzi et al., 2001; Baer & Martinez, 2006). The association between CM and patterns of attachment has repeatedly been reported within the trauma literature. It has been demonstrated that maltreated children (Haskett et al., 2006) and formerly abused adults are more likely to show a higher proportion of insecure or unresolved attachment (Muller et al., 2000; Weinfield et al., 2000; Muller, 2009; Bakermans-Kranenburg & van IJzendoorn, 2009; Buchheim et al., 2017; 2018; Buchheim & Diamond, 2018). According to the metanalysis of van IJzendoorn & Bakermans-Kranenburg (2008) clinical groups with internalizing and externalizing disorders predominantly consisted of individuals with insecure attachment patterns. In line with this, depressive symptomology has specifically been linked to insecure-preoccupied and unresolved attachment representations (Cooke et al., 2019; van IJzendoorn & Bakermans-Kranenburg (2008), Bauriedl-Schmidt et al., 2017; Buchheim et al., 2018). Recent research demonstrates that individuals with an insecure attachment seem particularly at risk to develop mental distress in less stressful times like a pandemic, but results are heterogenous. Moccia et al. (2020) found that confidence and discomfort with closeness were protective during the early phase of COVID-19 pandemic. In contrast Linag et al. (2021) suggested that parents with dismissing and fearful attachment styles and their children may be at higher risk during the COVID-19 pandemic and they should be given long-term attention. Results with respect to attachment styles during COVID-19 pandemic seem to be inconsistent, and insecure attachment patterns are considered to be related to maladaptive emotion regulation (Mikulincer et al., 2003).

Since depressive symptoms were repeatedly recorded throughout the whole project, this study also focused on depressive symptoms. In addition, studies have already found more than a threefold increase in depressive symptoms since the onset of the SARS-CoV-2 pandemic, so consideration of these symptoms is considered reasonable (Ettman et al., 2020). We may conclude that the current pandemic presents a great challenge to coping with pandemic-associated consequences, especially for individuals with a background of insecure or unresolved attachment. Moreover, we assume that the risk of developing depressive symptoms is increased compared to individuals with lower emotional burden due to the pandemic situation.

In view of the fact that numerous findings confirm the connection between the mental health of parents and their children (Fitzsimons et al., 2017; van der Waerden et al., 2015; Goodman et al., 2011), children of parents with an insecure or unresolved attachment representation seem particularly at risk of developing mental distress themselves especially in times of a pandemic. Therefore, it is necessary to understand the developmental pathway between the parental attachment representation of mothers with and without stressful early life experiences such as CM and their own as well as their children’s subsequent mental and behavioral outcomes in order to support positive child development, particularly in the context of additional stressors caused by the ongoing pandemic.

Therefore, the aim of our study was to analyze the interplay between the maternal attachment representation and the mothers’ and children’s mental health before and during the SARS-CoV-2-pandemic.

We hypothesized that mothers with insecure or unresolved attachment representations are more likely to report more severe depressive symptoms and that a lower quality of maternal mental health is associated with increased mental distress of children during the SARS-CoV-2 pandemic. We also assumed that the maternal history of CM is associated with maternal attachment representation.

## Methods

### Study Design

The data were assessed as part of the ([blinded]) joint interdisciplinary project. In a prospective study design, the joint project ([blinded]) examined protective and risk factors with regard to transgenerational transmission of maternal maltreatment experiences by including on psychological, biological, and social factors.

The interdisciplinary joint project ([blinded]) was funded by the ([blinded]) (October 2013–March 2017) from 2013 to 2017 and consists of five sub-projects, four clinical projects and an animal model. The study was approved by the Ethics Committee of ([blinded]) University, performed in accordance with relevant guidelines and regulations.

Mothers and their children were accompanied and examined throughout the child’s first years of life. All mother–child-dyads were recruited at the maternity unit of the ([blinded]) University Hospital. In the first step, the examinations included a screening one to three days postpartum (measurement time t0) to indicate maternal experiences of CM using the German version of the Childhood Trauma Questionnaire (CTQ) (Bader et al., 2009; Bernstein et al., 2003). This was followed by a first follow-up three months postpartum (t1), a second follow-up twelve months postpartum (t2), and a third follow-up about 3 years after the birth. In order to examine the current stress load experienced by families due to the current SARS-CoV-2-pandemic, all participating mothers were asked to take part in an online “SARS-CoV-2 pandemic survey”, which was available from May 18th – July 31st, 2020.

### Participants

Over a period of time from October 2013 to December 2015, 533 mother-child-dyads were recruited in the women’s hospital of the University Hospital of ([blinded]) shortly after childbirth. The criteria for participants to be included in the study were the mother’s age ≥ 18 years, sufficient knowledge of German and the state of health of mother and child. Exclusion criteria were poor health of the mother (e.g., AIDS disease, hepatitis, etc.), current or former drug or alcohol abuse by the mother, severe mental illness of the mother, serious birth complications, premature birth (less than 37 weeks of pregnancy) or an extremely low birth weight of the child (less than 1500 g). A total of 240 mothers provided written informed consent and were invited for data assessment 3 months postpartum (t1: laboratory and home visit). 158 mother-child-dyads participated in a further laboratory and home visit around 12 months of child’s age (t2) as well as around the child’s third birthday (t3). As part of a current data collection underway (t4), mothers were contacted again via mail if they wanted to participate in our online “SARS-CoV-2 pandemic survey” to study the impact of the SARS-CoV-2 pandemic on families and their children. 92 of the 158 participating mothers agreed to fill out the survey and completed it until the end of July 2020. Reasons for non-participation in the survey included time constraints, not reaching mothers, and unwillingness to participate in research specific to SARS-CoV-2. As the basis for all calculations, we only considered complete data sets of mother-child-dyads. Due to missing values for one case, we will report on a data set of *N* = 91.

Mothers were between 31 and 46 years old at the time of the “SARS-CoV-2-pandemic survey” (mean 38.14 years [SD 4.08 years]). The children’s age varied between 5 and 7 years (mean 6.03 years [SD 0.61 years]). Only marginally more boys (52.7%) than girls were included in the sample. The overwhelming majority reported to be of German origin (89.6%) and have a partner (95.7%). The distribution of the mothers’ education level showed that this sample was characterized by a high education exemption: 60.9% of mothers had completed a degree of higher education, 15.2% had attended 13 years of school education, 17.4% had achieved a basic secondary school degree (10 years of school education) and only 6.5% of participants had attended school for than 9 years or less. 6.5% of participants indicated a decrease in income and 26.1% indicated to work short time since the beginning of the SARS-CoV-2 pandemic. All descriptive statistics are presented in Table 1.

**Table 1:** Descriptive statistical data

Variable	*M*	SEM	SD	Median	Min	Max	Skewness	Kurtosis	%
Children’s age	6.03		0.61		4.98	7.14			
Girls									47.3
Boys									52.7
Age of mother	38.14	0.43	4.08	38.00	31.00	46.00	0.12	−0.69	
German citizenship									89.6
Partnership									95.7
Education									
University degree									60.9
Grammar school degree									15.2
Basic secondary school degree									17.4
No secondary school degree									6.5
Decrease in income during the pandemic									6.5
Short-time work during the pandemic									26.1
CM	34.15	1.29	12.35	30.00	25.00	81.00	2.17	4.37	
Maternal attachment representation									
secure									35.9
insecure									64.1
Before the pandemic									
Maternal depression	14.39	0.37	3.57	14.00	9.00	30.00	1.07	2.86	
Children’s mental health	11.54	0.29	2.73	11.00	2.00	18.00	0.34	0.91	
Emotional Problems	3.60	0.11	1.06	3.00	0.00	6.00	0.80	1.54	
Externalizing behavioral problems	5.33	0.13	1.27	5.00	2.00	8.00	0.36	−0.44	
Hyperactivity/in-attention problems	2.60	0.10	0.98	2.00	0.00	6.00	0.87	0.80	
During the pandemic									
Maternal depression	16.07	0.53	5.04	16.00	9.00	30.00	0.68	−0.05	
Children’s mental health	13.13	0.42	4.00	12.00	2.00	24.00	0.66	0.25	
Emotional problems	4.30	0.19	1.81	3.00	0.00	9.00	0.95	0.37	
Externalizing behavioral problems	5.97	0.19	1.77	5.00	2.00	10.00	0.60	−0.48	
Hyperactivity/attention problems	2.93	0.14	1.80	2.00	0.00	6.00	1.09	0.44	

### Measures

Maternal CM was assessed one to three days after the birth of the child with the German short version of the CTQ (Bader et al., 2009; Bernstein et al., 2003). This is a standard tool in the form of a retrospective self-report questionnaire for recording the experiences of physical, sexual and emotional abuse, as well as physical and emotional neglect before the age of 18 years (Wingenfeld et al., 2011). The CTQ assesses each subscale with five items on a 5-point Likert scale whereby the subscale scores range from 5 to 25 and the sum scores from “none” maltreatment experiences (25 points) over “minimal” to “extreme” maltreatment load (125 points). The sum score of all CTQ subscales was developed as a measure for the maltreatment load (Bernstein & Fink, 1998). The CTQ showed very good internal reliability in validation studies with Cronbach’s alpha between 0.79 and 0.94 (Bernstein et al., 1994).

To assess the attachment representation of the mothers in adulthood we used the Adult Attachment Projective Picture System (AAP) at the first follow-up (t1) three months after the children’s birth. The AAP is an objective, reliable and valid interview procedure in which the interviewee is presented with a set of picture stimuli which includes eight drawings consisting of one neutral warm-up picture and seven attachment scenes (George & West, 2012). The drawings show attachment situations on which individuals are alone or in potential attachment dyads. The AAP is based on the analysis of “story” responses to a set of 7 theoretically-derived attachment-related drawings of scenes depicting solitude, illness, separation, death and potential maltreatment. Individuals are asked to tell a story to each picture following a standardized set of interview probes. The classification is derived by evaluating the response patterns for the whole set of seven picture stimuli, each response of which is evaluated for content, discourse, and defensive processes. The interviews were performed by trained psychologists and audio-recorded to be evaluated according to defined criteria according the manual (George & West, 2012). The AAP assesses the four established attachment classifications: secure-autonomous (F), insecure-dismissing (Ds), insecure-preoccupied (E) and the unresolved attachment status (U). For our present study, the attachment representation of the mothers was divided into two major classifications: secure and an insecure attachment representation. The insecure attachment representation was comprised of attachment status Ds, E, and U.

The extent of the mothers’ depressive symptomology as an indicator of the mother´s mental health was assessed using the nine items of the depression module of the Patient Health Questionnaire (PHQ-9). This questionnaire was included in the “SARS-CoV-2 pandemic survey”, asking for an assessment of each item before the pandemic and during the pandemic. Each item can be answered on a 3-point Likert scale from “0” (not at all) to “3” (nearly every day). The screening instrument for diagnosing depression was developed for routine use in the somatic medical field. The screening for depression records one of the nine DSM-IV criteria for the diagnosis of “major depression” with each question. The evaluation of the PHQ-9 can, on the one hand, take place categorically and, on the other hand, it can also be interpreted in terms of the total value. The interpretation of the total value serves to assess the severity of the depression (Kroenke et al., 2001; Martin et al., 2006). This questionnaire has very good reliability with Cronbach’s alpha of 0.89 (Rief et al., 2004).

The psychological stress on children caused by the current pandemic was determined with the help of selected items from the German version of the Strengths and Difficulties Questionnaire (SDQ) (Klasen et al., 2003), an established short behavioral screening questionnaire rated by parents. Cronbach’s alpha is in the satisfactory range with a value of 0.7 (Muris et al., 2003). This questionnaire was also collected as part of the online survey, and the aim was here to evaluate the children´s mental health status before and during the pandemic in each case. Within the screening, positive and negative behavioral attributes of children including both strengths and difficulties are recorded on 5 subscales. The screening consists of 25 items, each with 5 items per scale: emotional problems, externalizing behavioral problems, hyperactivity/attention problems, problems with peers, and prosocial behavior to distribute. The answers were given on a three-point Likert scale (0 = not applicable, 1 = partially applicable, 2 = clearly applicable). To record children’s mental health as part of the “SARS-CoV-2-pandemic survey“ we selected the subscales concerning “emotional problems”,“ externalizing behavioral problems” and “hyperactivity/attention problems”. Due to the pandemic-associated restrictions in the context of school and kindergarten closures as well as the limitation of social contacts outside the family, we decided not to include the scales “problems with peers” and “prosocial behavior”.

The online survey also recorded other aspects such as the use of coping strategies, but these are not relevant to this study and are therefore not explained in more detail. Overall, all questions appeared first regarding the child, then regarding the mother, in order to avoid a constant change in the focus of attention.

### Statistical Analyses

We conducted our statistical analyses in IBM SPSS Statistics Version 27 for MacOS. Descriptive statistics were computed to examine variables’ distributions and characteristics. Normal probability plots and the results of the Kolmogorov-Smirnov-test graphically and statistically revealed that only our control variables and none of our model variables were normally distributed. However, the procedure we employed for our mediation analyses is based on bootstrapping and, thereby, can be considered robust against violations of the normal distribution assumption, according to Hayes (2018). Similarly, the analyses of variance we performed have been found to be relatively robust against violations of the normal distribution assumption (Schmider et al., 2010; Blanca et al., 2017). Additionally, we applied bootstrapping as a countermeasure against the consequences of not normally distributed variables when we computed the binary logistical regression analysis.

We inspected homoscedasticity of residuals and linearity graphically with residual scatterplots, which did not indicate any conspicuous deviations from homoscedasticity or linearity. Furthermore, the modified Breusch-Pagan-test did not statistically reject the assumption that the error variance was the same (*χ*²_before_(1) = 0.79, *p* = 0.38 and *χ*²_during_(1) = 0.34, *p* = 0.56). Therefore, we did not apply a heteroscedasticity consistent standard error estimator when performing the mediation analyses. Multicollinearity was assessed with the variance inflation factor and tolerances (VIF_before_ = 1.045, tolerance 0.957, and VIF_during_ = 1.086, tolerance = 0.921), which were located well within the limits acceptable for regression analysis and did not express any concern for multicollinearity. As part of our preliminary analyses, we performed a bootstrapped binary logistic regression analysis to test our hypothesis that the attachment representation of mothers in adulthood is predicted by the degree of their CM.

Furthermore, we reviewed the bivariate associations between our model and control variables by computing Pearson correlations. Two two-way mixed analyses of variance were conducted to inferentially test differences between mothers’ severity of depression and children’s mental health problems before and during the pandemic, respectively. Moreover, we tested group differences between mothers endorsing an insecure attachment relationship and mothers endorsing a secure attachment relationship as well as group differences between children with mothers endorsing either an insecure or secure attachment relationship.

We used the PROCESS macro for SPSS in version 3.5 to test two single mediator models (model 4) as described in the second edition of Hayes’ Introduction to Mediation, Moderation, and Conditional Process Analysis (2018). This procedure is a tool for modeling observed variables ordinary least squares and multiple regression path analyses. To test the total, direct and indirect effects inferentially, bootstrapped (*n* = 10.000) and bias-corrected confidence intervals were computed. We performed mediation analyses to test the total, direct and mediated effect of maternal attachment relationship via the maternal depressive symptomology before and during the pandemic on the children’s mental health problems overall as well as their emotional problems, externalizing behavioral problems and hyperactivity/attention problems, specifically, before and during the pandemic.

## Results

### Descriptive Analyzes

The observation of the histogram of the variable observing the level of depression experienced before the onset of the pandemic, revealed a right skewed and peaked distribution. This is underlined by the descriptive statistics. With a range between 9.00 and 33.00, the mean of 14.39 (SD = 3.57) showed that on average mothers reported a relatively low severity of depression before the onset of the pandemic. In contrast, the distribution of depression experienced during the pandemic graphically showed much greater variability within the same range and a less pronounced peak and right skewedness. Both its mean (*M* = 16.07, SD = 5.04) and median (MD = 16.00) descriptively points towards a comparably higher severity of depression during the pandemic.

The children’s mental health before the onset of the pandemic averaged 11.54 (SD = 2.73) on a range between 2.00 and 18.00. Graphically, the children’s psychological symptoms during the pandemic varied considerably more on a wider range with a maximum of 24.00. Its mean of 13.13 (SD = 4.00) descriptively reflected an increase in children’s mental health problems according to the report of their mothers. Both variables were slightly right skewed with fewer values towards the higher end of the range. An outlier below Q1 could be detected but did not distort either distribution noteworthily and therefore, was not excluded. The analysis of the subscales of SDQ, emotional problems, externalizing behavioral problems and hyperactivity/attention problems, respectively, and the comparison between the children’s specific mental health problems before and during the pandemic followed similar distribution patterns.

35.9% of mother had been classified to have a secure attachment pattern and 64.1% of mothers endorsed an insecure (including Ds, E, and U). The CM experienced by mothers ranged between a minimum of 25.00 and 81.00. With regard to this, the mean (*M* = 34.15, SD = 12.35) and the median (MD = 30.00) illustrate that at least half of all participating mothers indicated a relatively low sum of CM. Inspection of the histogram demonstrated a pronounced right skewness as well as a noticeably peaked distribution. Regarding the higher end of the range, analysis of the boxplot revealed four outliers more than 1.5 times above Q3 and six extreme values more than 3 times above Q3. We decided not to exclude them as they provided essential variability in the sample.

### Regression Analysis, Pearson Correlations, Two-Way Mixed Analyses of Variance

#### Binary logistic regression analysis

Results of the binary logistic regression analysis are presented in Table 2. The model reached significance (*χ*^2^(1) = 8.29, *p* = 0.004) and a significant association between the mothers’ history of CM and their attachment representation (*χ*^2^(1) = 5.02, *p* = 0.002) could be found.

**Table 2:** Binary logistic regression analysis

	df	Wald χ2	Bootstrapped B	Bootstrapped SE	Bootstrapped CI	*p*
Maternal CM	1	5.02	0.73	0.03	0.036–0.148	0.002

#### Zero-order bivariate correlations

Before conducting the mediation analysis, we computed Pearson correlations in order to review the bivariate associations between variables, which are presented in Table 3. Attachment representation significantly correlated with the maternal depression level before (*r*(89) = 0.23, *p* < 0.05) and during the pandemic (*r*(89) = 0.23, *p* < 0.05) whereas it was not significantly related to the children’s mental health before (*r*(89) = 0.08, *p* > 0.05) and during the pandemic (*r*(89) = 0.11, *p* > 0.05). The moderate association between maternal depression severity and children’s mental health before the pandemic (*r*(89) = 0.34, *p* < 0.01) and the strong association between maternal depressive symptomology and children’s mental health during the pandemic (*r*(89) = 0.57, *p* < 0.01) were found to be highly significant. The maternal depression level before the pandemic was also moderately and significantly correlated with the children’s mental health during the pandemic (*r*(89) = 0.37, *p* < 0.01).

**Table 3:** Pearson correlations between model variables

Variable	1	2	3	4	5	6
1 Attachment representation	1	0.26*	0.23*	0.30**	0.08	0.11
2 CM		1	0.43**	0.37**	0.06	0.09
3 Depression before the pandemic			1	0.66**	0.34**	0.37**
4 Depression during the pandemic				1	0.42**	0.57**
5 Children’s mental health before the pandemic					1	0.73**
6 Children’s mental health during the pandemic						1

*p < 0.05

**p < 0.01

The maternal depression severity before the pandemic was significantly and moderated correlated with children’s emotional (*r*(89) = 0.32, *p* < 0.01) and externalizing behavioral problems (*r*(89) = 0.38, *p* < 0.01), whereas it was not significantly related to children’s hyperactivity/attention problems (*r*(89) = 0.14, *p* > 0.05) before the pandemic. In contrast, the mothers’ depressive symptomology during the pandemic showed a significant moderate to strong association with children’s emotional problems (*r*(89) = 0.45, *p* < 0.01), externalizing behavioral problems (*r*(89) = 0.49, *p* < 0.01) and hyperactivity/attention problems (*r*(89) = 0.43, *p* < 0.01), respectively, during the pandemic. The link between the mothers’ extent of depressive symptoms before the pandemic and children’s different types of mental health problems during the pandemic was insignificant for children’s emotional problems (*r*(89) = 0.20, *p* > 0.05) and only small for children’s externalizing behavioral problems (*r*(89) = 0.27, *p* < 0.01) as well as hyperactivity/attention problems (*r*(89) = 0.23, *p* < 0.01).

In this context, it is necessary to consider that, as Bollen (1989) has stated, “correlation is neither necessary nor sufficient for causation” (p. 52). Contemporary recommendations for mediation analysis emphasize that a significant bivariate link between the predictor and criterion is not a conclusive requirement for the potential existence of a mediating effect, contrasting Baron & Kenny (1986)’s widely popularized criteria to determine mediation based on their causal steps approach.

#### Two-way-mixed analyses of variance

The first two-way mixed analysis of variance revealed a significant main effect of the pandemic on the maternal depression level experienced according to the mothers’ self-report, *F*(1, 87) = 11.72, *p* < 0.001. Drawing on the descriptive statistics, the severity of depressive symptoms during the pandemic was found to be significantly higher than before the onset of the pandemic. Furthermore, the main effect of maternal attachment representation on depression severity was significant, *F*(1, 87) = 7.01, *p* < 0.05. Therefore, mothers with an insecure attachment representation significantly differed in the severity of their depressive symptomology before and during the pandemic from mothers with a secure attachment representation. As the descriptive mean differences demonstrate, mothers endorsing an insecure attachment tended to experience more severe depressive symptoms compared to mothers endorsing a secure attachment, especially during the pandemic.

The second two-way mixed analysis of variance also pointed out a significant main effect of the pandemic on the children’s mental health problems, *F*(1, 87) = 26.11, *p* < 0.001. Therefore, in line with the descriptive statistics, the children’s mental health problems, according to the mothers’ report, showed a significant increase since the onset of the pandemic. However, the main effect of an insecure, as opposed to a secure maternal attachment, did not reach significance, *F*(1, 87) = 0.96, *p* = 0.33, whereby children whose mothers endorsed an insecure attachment did not significantly differ in their mental health problems from children whose mothers endorsed a secure attachment. The results of both two-way mixed analyses of variance are presented in Table 4.

**Table 4:** Pearson correlations between model variables and the variables based on the children’s mental health

Variable	1	2	3	4	5	6	7	8	9	10
1 Attachment representation	1	0.26*	0.23*	0.30**	0.04	−0.03	0.09	0.12	0.05	0.15
2 CM		1	0.43**	0.37**	0.03	0.04	0.07	0.03	0.05	0.15
3 Maternal depression before			1	0.66**	0.32**	0.20	0.38**	0.27**	0.14	0.23*
4 Maternal depression during				1	0.39**	0.45**	0.37**	0.49**	0.29**	0.43**
5 Emotional Problems before					1	0.67**	0.67**	0.54**	0.40**	0.33**
6 Emotional Problems during						1	0.42**	0.65**	0.20	0.35**
7 Externalizing behavioral problems before							1	0.69**	0.47**	0.39**
8 Externalizing behavioral problems during								1	0.35**	0.45**
9 Hyperactivity/attention problems before									1	0.74**
10 Hyperactivity/attention problems during										1

*p < 0.05

**p < 0.01

### Mediation Analysis

#### Model 1: Mediation analysis before the SARS-CoV-2 pandemic

**Fig. 1:**
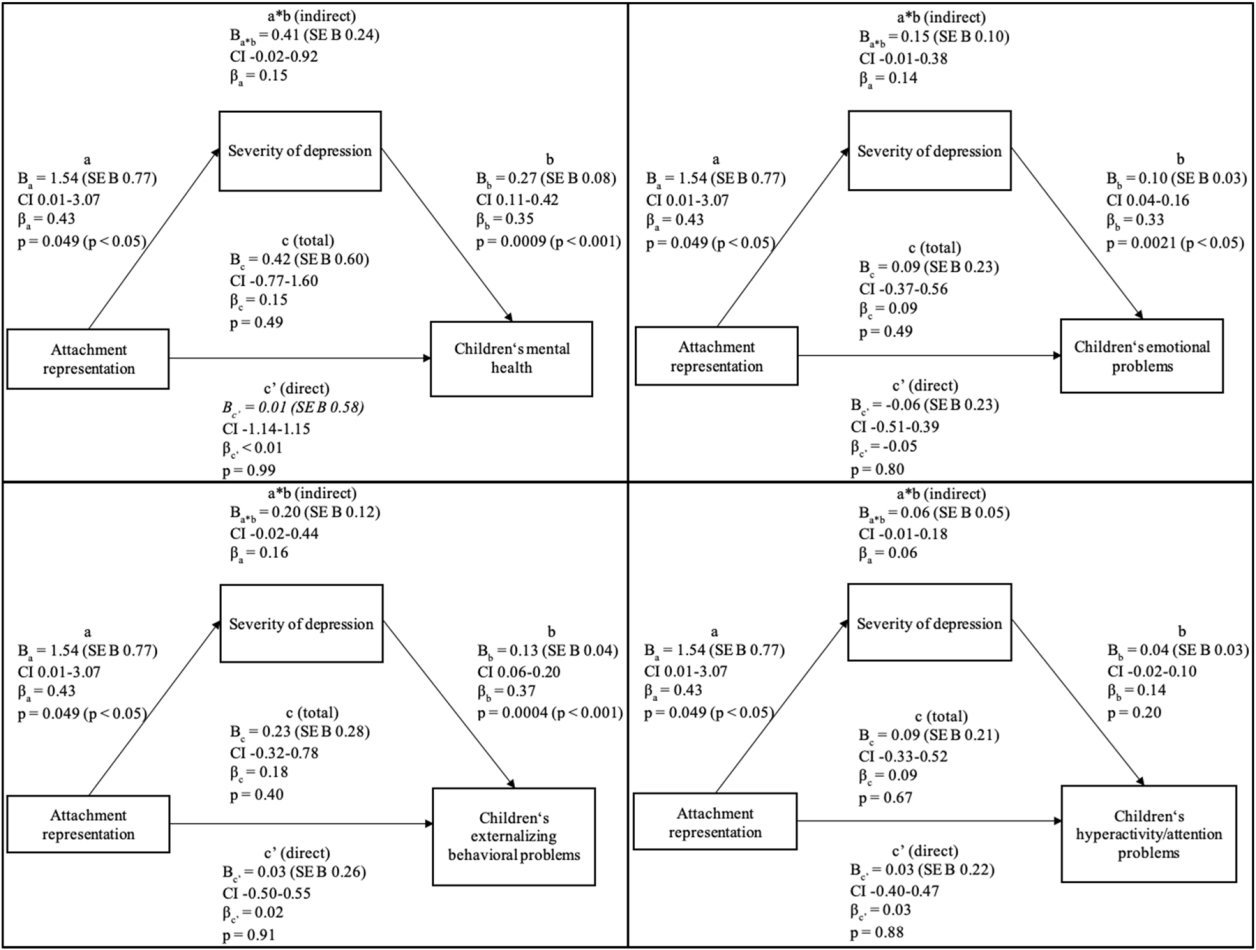
Model 1: Proposed mediation of maternal attachment representation and children’s mental health problems through the maternal severity of depression before the SARS-CoV-2 pandemic

The mediation analysis found that neither the total (*p* = 0.49) nor the direct (*p* = 0.99) or indirect (95% bootstrap CI -0.02-0.92) effects were significant. Unstandardized coefficients with corresponding standard errors, standardized coefficients, confidence intervals, and p values of the first mediation analysis are presented in Fig. 1.

As indicated by the bivariate correlations, attachment representation significantly predicted the maternal depression severity experienced before the onset of the pandemic (*B*_a_ = 1.54, SE *B*_a_0.77, *p* < 0.05). Mothers with an insecure attachment were more likely to have more severe depressive symptoms. The mothers’ depressive symptomology, in turn, significantly predicted the children’s mental health before the pandemic (*B*_b_ = 0.27, SE B_b_0.08, *p* < 0.001). This corresponds with the highly significant zero-order correlation indicating that children whose mothers struggled with depressive symptoms were more likely to exhibit mental health problems. However, the mediating effect was not significant as zero was included in the bootstrapped confidence interval (*B*_a*b_ = 0.41, SE B_a*b_0.24, CI -0.02 to 0.92) and could at best be argued to be marginally significant. As the preliminary analysis of bivariate correlations indicated, the total effect of maternal attachment representation on children’s mental health did not reach significance (*B*_c_ = 0.42, SE *B*_c_ = 0.60, *p* > 0.05). Furthermore, under consideration of the potentially intervening variable, no significant direct influence of mothers’ attachment representation on children’s mental health was found (*B*_c’_ = 0.01, SE *B*_c’_0.58, *p* > 0.05). Therefore, whether mothers endorsed a secure or an insecure attachment relationship did not directly affect children’s mental health (Table 5).

**Table 5:** Results of two-way mixed analyses of variance

	df	*F*	*p*
Maternal severity of depression			
Before vs. during the pandemic	1	11.716	<0.001
Secure vs. insecure attachment relationship	1	7.022	0.010
Error	87		
Children’s mental health problems			
Before vs. during the pandemic	1	26.114	< 0.001
Secure vs. insecure attachment relationship	1	0.964	0.329
Error	87		
	**Attachment**	***M***	**SD**
Severity of depression before the pandemic	secure	13.39	3.77
	insecure	14.95	3.36
Severity of depression during the pandemic	secure	14.15	4.41
	insecure	17.14	5.08
	**Attachment**	***M***	**SD**
Children’s mental health problems before the pandemic	secure	11.27	2.64
	insecure	11.69	2.80
Children’s mental health problems during the pandemic	secure	12.58	3.72
	insecure	13.45	4.14

When controlling for age of mother, education of mother, age and gender of the child, the same pattern of results remained with total, direct and indirect effects not significant.

Moreover, the same pattern of results was revealed for children’s emotional problems, externalizing behavioral problems, and hyperactivity/attention problems before the pandemic as the total, direct and indirect effects were not significant.

#### Model 2: Mediation analysis during the SARS-CoV-2 pandemic

**Fig. 2:**
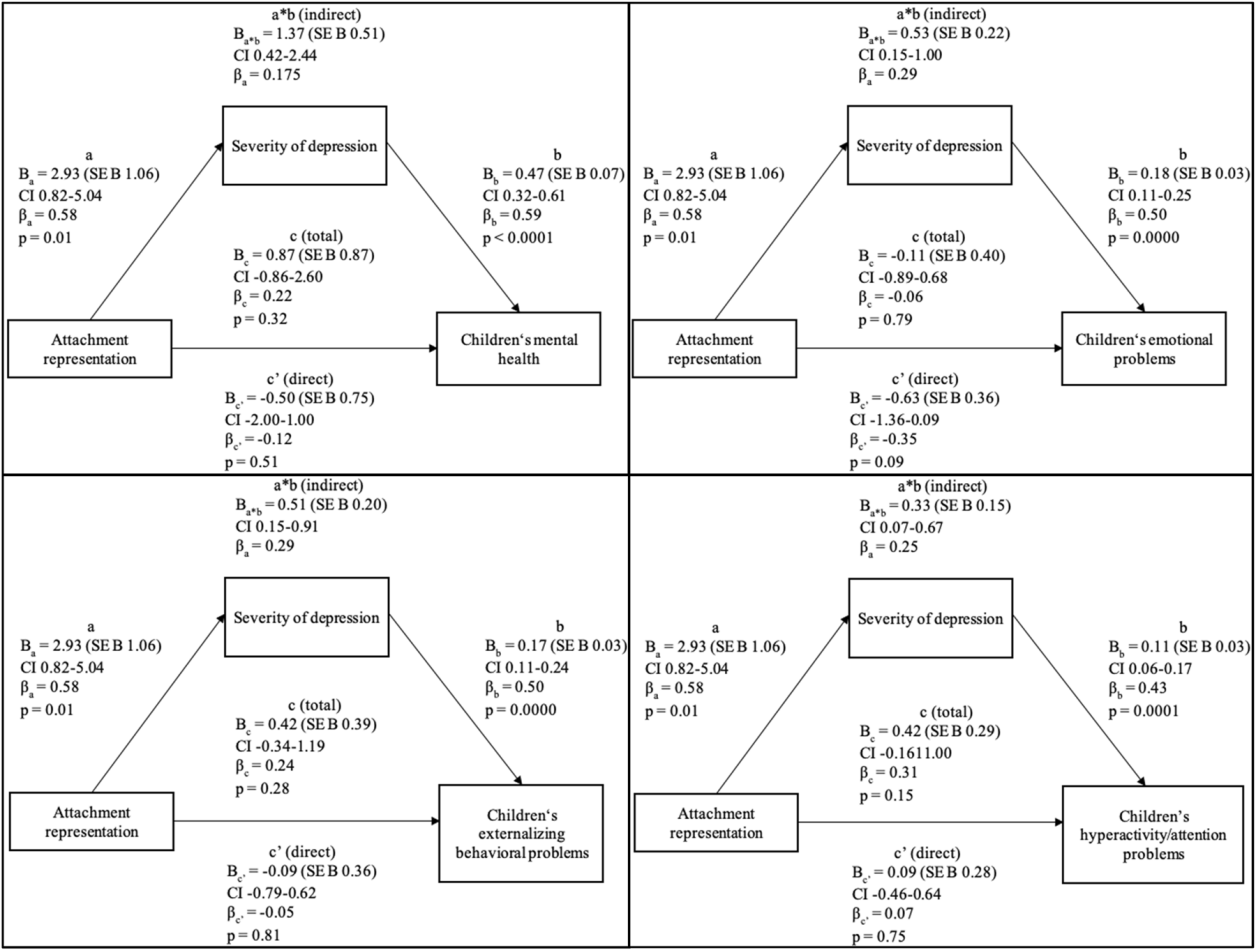
Model 2: Proposed mediation of maternal attachment representation and children’s mental health problems through the maternal severity of depression during the SARS-CoV-2 pandemic

The total (*p* = 0.32) and direct effect (*p* = 0.51) did not reach significance. However, the results suggested the severity of depression experienced by mothers during the pandemic to significantly and fully mediate the relationship between their attachment representation and children’s mental health during the pandemic (bootstrapped CI 0.42–2.44). Unstandardized coefficients with corresponding standard errors, standardized coefficients, confidence intervals and p values of the second mediation analysis are presented in Fig. 2.

Maternal attachment representation was found to significantly predicted the mothers’ depression symptomology during the pandemic (*B*_a_ = 2.93, SE *B*_a_ 1.06, *p* < 0.05), which significantly affected their children’s mental health during the pandemic (*B*_b_ = 0.47, SE *B*_b_ 0.07, *p* < 0.001). Therefore, mothers endorsing an insecure attachment tended to have more severe depressive symptoms, which, in turn, increased the likelihood for children’s mental health problems.

As indicated by preliminary zero-order correlations, no significant total effect of maternal attachment representation on children’s mental health during the pandemic was found (*B*_c_ = 2.93, SE *B*_c_ 1.06, *p* > 0.05). However, the depression symptomology of mothers during the pandemic was identified as a significant mediator between their attachment representation and their children’s mental health problems during the pandemic (*B*_a*b_ = 1.37, SE *B*_a*b_ 0.51, CI 0.42–2.44). Under consideration of the mediator, no significant direct association between maternal attachment representation and children’s mental health was revealed (*B*_c_’ = –0.12, SE *B*_c_’ 0.75, *p* > 0.05). Therefore, the results are consistent with a full mediation as the direct effect was not significant when including the mediator into the analysis.

When controlling for age of mother, education of mother, age and gender of child, the indirect effect of the maternal severity of depressive symptoms fully mediating between the mothers’ attachment representation and their children’s mental health problems remained significant (*B*_a*b_ = 1.37, SE *B*_a*b_ = 0.51, CI 0.42–2.44).

It may appear counterintuitive to infer that an indirect effect exists in the absence of a total effect. Such an opposing conclusion may stem from the assumption that X has no effect on Y if the path c is not statistically significant or zero whereas contemporary methodologists argue that mediation can take place without evidence of a total effect of X on Y (Hayes & Rockwood, 2017). Therefore, it is substantively useful to consider contemporary recommendations and practices for mediation analysis as offered by Hayes & Rockwood (2017) to clear up such misconceptions. As they clarify, an insignificant total effect in a mediation model implies that no linear association can be detected on aggregate when all paths influencing the relationship between X and Y are added (Hayes & Rockwood, 2017).

The same pattern of results was shown for children’s emotional problems, externalizing behavioral problems and hyperactivity/attention problems during the pandemic as the mediating effect remained significant alongside an insignificant total and direct effect for all criteria.

## Discussion

The ongoing pandemic has proven to have far-reaching and exceptional implications for children, adolescents and their families from an attachment perspective (Steele, 2020). The social-distancing restrictions implemented into daily life to contain the spread of the SARS-CoV-2 virus, such as school closures, home schooling (UNESCO), limited private shielding, quarantine as well as the cancellation of out-of-home leisure time activities (Wang et al., 2020a; Wang et al., 2020b; Flaxman et al., 2021) have added to the already existing burden of family situations, potentially to the point of overtaxing their capacity to handle the related challenges (Fegert et al., 2020). Furthermore, it has to be considered that external support provided by other family members and institutional social systems failed (Fegert et al., 2020).

According to current research examining the pandemic-related effects on young children between 3 and 6 years of age, they are significantly more likely to be affected by stress symptoms in their emotional and social development compared to older children (Viner et al., 2020). This underlines the importance of a stable and secure family environment, which is fundamentally formed by mentally healthy parents, as a strong protective factor for children in their daily life (Schofield et al., 2013; Flouri et al., 2015; Hostinar et al., 2014; Yue et al., 2020). Therefore, our study aimed to investigate for the first time maternal mental health under consideration of mothers’ attachment influenced by their history of CM and previous relationship experiences by contrasting the effect of these factors on the children’s mental health before and during the SARS-CoV-2-pandemic. Our results corroborate and expand on previous findings not only by examining the associations between these variables but by modelling their interplay in a mediation analysis. Understanding the mechanism behind the influence of maternal insecure attachment on their children’s mental health provides important information to derive further clinical and practical implications.

Several previous studies indicate that adult participants with experiences of CM are more likely to develop an insecure and unresolved attachment representation in adulthood (Bakermans-Kranenburg & van IJzendoorn, 2009; Muller, 2009; Muller et al., 2012). Moreover, Bailey and colleagues (2007) observed in their sample with at-risk adolescent mothers that a general history of CM was related to an unresolved attachment. Complementing these findings, our results show that maternal current attachment representations were significantly predicted by and related to the extent of the CM they had experienced.

Muller and colleagues (2012) further identified attachment representation as a relevant mediator between CM and adult psychopathology. Their findings showed that insecure attachment was predicted by psychological and physical abuse and predicted externalizing, internalizing and trauma-related symptomology (Muller et al., 2012). Other studies demonstrated that depressive symptomology was associated with insecure-preoccupied and unresolved attachment representations (Dagan et al., 2018; Chow & Ruhl, 2014, Bauriedl-Schmidt et al., 2017). In accordance with this finding, we found in our study that maternal attachment representation significantly related to the severity of depressive symptoms experienced by mothers both before and during the pandemic. Mothers with an insecure attachment representation significantly differed in their depression level before and during the pandemic from mothers with a secure attachment representation. Based on the descriptive mean difference, mothers with insecure or unresolved attachment representations tended to report more severe symptoms of depression and this tendency was especially clear during the pandemic. This is in line with the findings by Hankin (2005) who reported of a significant association CM, insecure attachment and depressive symptoms in late life (Hankin, 2005). Widom and colleagues (2018) recently confirmed that individuals with a history of CM are at higher risk for an insecure or unresolved attachment in adulthood and higher levels of depression as well as lower levels of self-esteem.

Furthermore, several studies have suggested an association between the mental health of parents and their children (Fitzsimons et al., 2017; van der Waerden et al., 2015; Goodman et al., 2011; Reger et al. 2020; Roos et al. 2021). Fitzsimons and collegues (2017) observed parental mental health as the strongest correlate of children’s mental health even under consideration of other covariates. In a birth cohort study Waerden and collegues (2015) reported that children whose mothers indicated a persistent trajectory of depressive symptoms showed the greatest levels of emotional and behavioral difficulties. Consistent with this finding, we found that the maternal depression level significantly correlated with their children’s mental health problems before and during the pandemic. Here, the children’s mental health problems were likely to be more pronounced when their mothers experienced more severe depressive symptoms. This association was, in fact, noticeably stronger during the pandemic compared to the moderate tendency before the onset of the pandemic. This is in line with our assumption that maternal mental health impacts their children’s mental health more strongly under the additional challenges and stress posed by the pandemic. Our descriptive and inferential results also outline a significant increase in the depressive symptomology experienced by mothers and in their children’s mental health problems during the pandemic compared to the mothers’ retrospective estimation of their depression level and their children’s mental health before the pandemic. This is in line with our assumption that the stress load of families in times of the ongoing pandemic increases persistently, and that, in turn, this pandemic-associated stress load enhances the risk to develop or amplify a depressive symptomology.

However, our study further aims to provide insight into how a maternal history of CM and the associated risk to develop and carry an insecure and unresolved attachment representation into adulthood significantly affects their psychopathology and their children’s mental health. Therefore, we have created a model to put into context the corresponding variables both before and during the pandemic. In view of the findings of our preliminary analyses, we did not expect a total or direct effect of mothers’ attachment representation on children’s mental health before or during the pandemic. Our present study did not observe a significant link between the maternal attachment representation and their children’s mental health and during the pandemic. Furthermore, children of mothers with an insecure attachment did not significantly differ from children of mothers with a secure attachment in their mental health problems across the assessment of their mental health before and during the pandemic. Instead, we set out focus on investigating the interplay of attachment representation, the mothers’ mental health struggles and their children’s emotional and behavioral difficulties. The relevance of our study regards the examination of how the effect of mothers’ attachment representation on their children’s mental health may operate in order to understand the underlying mechanism and to establish a framework for future research approaches.

The results of our mediation analyses suggest that the mothers’ depressive symptomology significantly and fully mediated the relationship between attachment representation and children’s mental health during the pandemic, while the total and direct effect, indeed, did not reach significance as expected based on our preliminary analyses. Therefore, during the pandemic, mothers with attachment insecurity were at a significantly higher risk to experience more severe symptoms of depression, which significantly contributed to their children’s emotional and behavioral difficulties. Furthermore, we found the mothers’ severity of depression to function as a significant mediator between the mothers’ attachment representation and children’s specific types of mental health problems. During the pandemic, the mothers’ attachment representation significantly predicted a higher level of depression-related symptoms, which was found to significantly increase the likelihood of children’s emotional problems, externalizing behavioral problems, and hyperactivity/attention problems, respectively.

This corroborates our assumption that a maternal history of CM and the associated attachment insecurity substantively influence children’s subsequent mental and behavioral outcomes through the joint association with the mothers’ depressive symptomology. Therefore, they should be considered as additional risk factors, not exclusive but particularly in times of paramount stress. However, we only observed the mothers’ depressive symptomology as a significant mediator during the pandemic. In contrast, our analyses did not reveal a significant indirect effect of maternal attachment representation on children’s mental health through the mothers’ severity of depression before the onset of the pandemic. Confirming our assumption based on the results of our preliminary analyses, no total or direct effect of maternal attachment representation on children’s mental health was found. The mothers’ attachment representation was significantly related to their depressive symptomology, which significantly predicted their children’s mental health problems, but the mediating effect was not significant. Therefore, it can, at best, be argued that a greater severity of depressive symptoms experienced by mothers marginally mediated the influence of attachment insecurity on children’s mental health problems. The same pattern applied to the mediating effect of mothers’ depressive symptomology between their attachment representation and their children’s specific types of mental health problems. Even though mothers with an insecure attachment were more likely to have more severe depressive symptoms, which appeared to significantly increase the likelihood for their children to exhibit emotional problems, externalizing behavioral problems and hyperactivity/attention problems, respectively, the indirect effect did not reach significance. The results showed that mothers with an insecure attachment were more likely to have more severe depressive symptoms which appeared to significantly increase the likelihood for their children to exhibit emotional problems, externalizing behavioral problems and hyperactivity/attention problems. Nevertheless, the indirect effect from mothers attachment representation to child’s mental health did not reach significance.

The findings highlight, especially in times of a pandemic, that possible risk factors with regard to the mental health of children must be extensively investigated. The analyses emphasize that the previous history of a parent as well as the stressors within in the family, such as the intelligence of the parents, may play a crucial role in how the family and especially the children within the family cope with times such as a pandemic in terms of health. Consequently, the evidence emphasizes the necessity to consider the unique needs and backgrounds of families with children and to offer support to cope with the current crisis. For example, families with a known parental history of CM could be offered regular support services to intervene early. Furthermore, our findings suggest that addressing mothers with insecure attachment and a history of CM with concurrent depressive symptomology might provide a fruitful approach for preventing the development of children’s internalizing and externalizing problems. Breaking the vicious circle of transgenerational transmission of maternal abuse experiences and inadequate parental behavior resulting in children’s attachment insecurity and adult psychopathology is essential and might be underlined by our findings. The development of appropriate psychoeducational measures about these relationships for affected mothers could also be helpful in this regard.

The present findings indicate that the effects of a pandemic such as the SARS-CoV-2- should be the target of future studies in the context of the family system. Fegert et al. (2020) already addressed the fact that the pandemic has particularly affected disadvantaged and marginalized children and adolescents and that especially vulnerable families will be exposed to numerous additional stress factors as a result of a possible economic recession, even after the pandemic (Fegert et al., 2020). Even if the current pandemic is both stressful and restrictive, it can contribute to the development of new innovative approaches, especially with regard to support and counseling offers for young families, which make it possible to support and accompany families more comprehensively (like such as increased telephone and online advice offers). The inclusion of other variables such as post-traumatic stress or resilience factors should also be undertaken in further studies on this topic to develop assistance programs for burdened families that are even better adapted to their needs.

We have to consider several limitations in the present study. First, our sample is limited to mothers of the birth cohort of mother-child dyads that had complete data sets and completed an online survey. Their willingness to participate in the survey may be accompanied by an openness to indicate their own depressive symptomology and their children’s mental health problems. Concurrently, it has to be considered that the ratings and answers regarding such questions may be affected by social desirability. Similarly, mothers retrospectively gave indications of their level of depression as well as their children’s mental health problems before the pandemic compared to their rating of their depressive symptoms and their children’s mental health within the last 2 weeks. Therefore, the assessment of recent symptoms and emotional or behavioral difficulties may have been more salient or may have been influenced by memory effects. The mental state of the mother may also have led to distorted assessments of their children´s mental health. Furthermore, our data collection was temporally limited to a short time period at the beginning of the pandemic, which focuses our study on a small sample. Therefore, future studies are needed to confirm our results and substantiate our proposed model with a larger sample size.

Second, our findings cannot be generalized to all families as our sample is characterized by a large number participants who were highly educated, had a partner and did not experience an decrease in income due to the pandemic, which cannot be seen as representative of the general public and can function as protective factors against detrimental consequences of CM. Meng et al. (2018) have reported higher education and socioeconomic status to be positively related to psychological well-being and life satisfaction. In addition, Ford (2012) observed that a lower educational level of women with history of victimization was associated with more severe trauma-related symptoms in their sample inferring that posttraumatic resilience may be promoted through educational attainment (Ford, 2012). Moreover, it is noteworthy that our sample included mothers with secure attachment representations despite a history of CM and that the majority of our sample reported to be live in a partnership. This may point towards the experiences of positive and successful relationships in adulthood buffering a history of adverse experiences (Mikulincer & Shaver, 2007). Furthermore, the overrepresentation of being in a partnership may have attenuated the lack of social support experienced by single parents in particular during the ongoing pandemic. The use of other variables, such as the number of children living in the household or workplace, should also be collected in future studies to further describe the participating families. It is possible that this will reveal other protective factors that the families exhibit and can therefore reduce the influence of CM. There could also be selective dropout of subjects: one of the most common reasons for non-participation was lack of time. We may thus not have been able to include mothers with a particularly high stress level in the study, which may have led to biased results.

Third, several studies, systematic reviews and meta-analyses have observed an increased likelihood of, for example, children’s emotional, behavioral, and adjustment difficulties associated with paternal mental health problems (Davé et al., 2008; Kane & Garber, 2009; Weitzman et al., 2011; Sweeney & MacBeth, 2016; Cheung & Theule, 2019). Therefore, we would like to point out that the importance of considering the paternal or second caregiver’s psychopathology on children’s mental health. Accordingly, interviewing additional family members such as the father could lead to further findings and should be implemented in future studies. Empirical statements clearly show the link between maternal depression and adverse outcomes for children. Investigations of associations between a parental symptomology and child development has not yet gained as much attention but should be included in an expanded model.

Also to be mentioned is the use of the PHQ-9 as a questionnaire to assess depressive symptomatology. This does not capture one symptom of depression, which is, however, necessary in the DSM-V for this diagnosis: namely, the restriction in various areas of life due to the symptoms described. Since the PHQ-9 is only intended to serve as a screening instrument for a quick overview and it is an established instrument in research, its use is not wrong, but should be supplemented in the future by other questionnaires regarding depression symptoms.

Last, as mentioned, only 6.5% of participating mothers indicated a decrease in income since the beginning of the pandemic and 26.1% reported to be working short time during the pandemic. However, our data collection took place at the beginning of the pandemic and, even though our sample cannot be considered representative of the general public, more people may have lost their jobs and experienced financial hardship over the course of the pandemic. As Fitzsimons and colleagues (2017) found, not only was current and persistent poverty related to an adverse mental health of children but also a transition into poverty. The aggravation of the ongoing pandemic has led to prolonged situations of lockdown and, thereby, has endangered even more jobs compared to the beginning of the pandemic. In view of this, the prospective trajectory of parents’ socio-economic situation may function as another risk factor to children’s mental health during and after the pandemic. In addition, other factors than the pandemic may have caused the change in the mother’s depressive symptomatology as well as the child’s mental health that were not considered in this study.

However, concludingly, our results give an insight into the relevance of considering and modeling the mechanism through which effects may operate. With our present study we might have been able to improve the understanding of the interplay between CM, parental attachment representations, adult symptomology, and children’s mental health.

## Conclusion

Our findings highlighted that, due to parental CM, maternal attachment representation can be considered as a risk factor for maternal and children’s mental health during a pandemic. The previous maternal attachment experiences seem to have a significant influence on how parents deal with stressful situations like a pandemic and seem to lead to a risk for developing depressive symptoms affecting the mental health of their children. This finding emphasizes the necessity to consider the unique needs of families with children and to offer support for coping with the current crisis.

## Data Availability

The datasets analyzed during the current study are available on a database of the University Hospital of (([blinded])).
